# miR‐145 attenuates phenotypic transformation of aortic vascular smooth muscle cells to prevent aortic dissection

**DOI:** 10.1002/jcla.23773

**Published:** 2021-11-12

**Authors:** Zhi‐Huang Qiu, Jian He, Tian‐ci Chai, Yu‐ling Zhang, Hao Zhou, Hui Zheng, Xiao‐song Chen, Li Zhang, Yu‐mei Li, Liang‐wan Chen

**Affiliations:** ^1^ Department of Cardiac Surgery Union Hospital Fujian Medical University Fuzhou China; ^2^ Department of Plastic Surgery Union Hospital Fujian Medical University Fuzhou China; ^3^ Department of Physiology and Pathophysiology School of Basic Medical Sciences Fujian Medical University Fuzhou China; ^4^ Department of Toxicology Fujian Center for Evaluation of New Drug Fujian Medical University Fuzhou China

**Keywords:** aortic dissection, mechanical strain, miR145, phenotype transformation, vascular smooth muscle cells

## Abstract

**Background:**

*miR*‐*145* is closely related to vascular smooth muscle cells (VSMC) phenotype transformation; however, the regulatory mechanisms through which *miR*‐*145* regulates the VSMC phenotype transformation under mechanical stretching are unclear. In this study, we evaluated the roles of *miR*‐*145* in VSMCs subjected to mechanical stretching in aortic dissection (AD).

**Methods:**

The expression of miR‐145 in the aortic vessel wall of model animals and patients with AD was analyzed by quantitative polymerase chain reaction. miR‐145‐related protein‐protein interaction networks and Wikipathways were used to analyze VSMC phenotypic transformation pathways regulated by miR‐145. We used gain‐ and loss‐of‐function studies to evaluate the effects of miR‐145 on VSMC differentiation under mechanical stretch induction and assessed whether Krüppel‐like factor 4 (KLF4) was regulated by miR‐145 in the aorta under mechanical stretch conditions.

**Results:**

*miR*‐*145* was abundantly expressed in the walls of the normal human aorta, but was significantly downregulated in animal models and the walls of patients with dissection. We found that contractile phenotype‐related proteins were downregulated in VSMCs subjected to mechanical stretching, whereas the expression of secreted phenotype‐related proteins increased. *miR*‐*145* overexpression also downregulated contractile phenotype‐related proteins in VSMCs and suppressed upregulation of phenotype‐related proteins. Finally, under mechanical stretching, KLF4 expression was significantly increased in VSMCs, and overexpression of *miR*‐*145* blocked this effect.

**Conclusion:**

Our results confirmed that mechanical stretch‐induced phenotypic transformation of VSMCs to promote AD via upregulation of KLF4; this mechanism was regulated by *miR*‐*145*, which directly modulated KLF4 expression and VSMC differentiation.

## Graphical abstract

*miR‐145* was abundantly expressed in the walls of the normal human aorta, but was significantly downregulated in animal models and the walls of patients with dissection. Contractile phenotype‐related proteins were downregulated in VSMCs subjected to mechanical stretching, whereas the expression of secreted phenotype‐related proteins increased. Mechanical stretch induced phenotypic transformation of VSMCs to promote AD via upregulation of KLF4. This mechanism was regulated by *miR‐145*, which directly modulated KLF4 expression and VSMC differentiation.

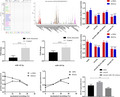

## 1 INTRODUCTION

Aortic dissection (AD) refers to tearing of the intima of the aorta, entry of blood into the middle layer of the artery wall, and peeling off of the intima to form a false cavity. Once rupture occurs, massive bleeding and death may result. Thus, AD is a catastrophic disease.^1^, ^2^, ^3^ However, the specific pathogenic mechanisms of AD have not yet been reported.

Vascular smooth muscle cells (VSMCs) are mainly located in the media of the aorta and play important roles in maintaining normal vascular morphology and biological functions.^4^ Many recent studies have reported that the phenotypic transformation of VSMCs is closely related to the occurrence and development of AD.^5^, ^6^, ^7^ Additionally, microRNAs (miRNAs) have been shown to regulate the phenotypic transformation of VSMCs under physiological and pathological conditions, thereby promoting VSMC differentiation and proliferation.^8^, ^9^, ^10^ In particular, *miR*‐*145* is closely related to VSMC phenotype transformation^11^; however, the regulatory mechanisms through which *miR*‐*145* regulates VSMC phenotype transformation under mechanical stretching are unclear, and few studies have evaluated VSMCs in patients with AD.

Accordingly, in this study, we collected aortic vessel walls from patients with acute AD and isolated and cultured high‐purity aortic VSMCs (AD‐VSMCs). We then simulated mechanical stretching caused by hypertension and studies the phenotypes of AD‐VSMCs subjected to various stretch conditions. Finally, we investigated the involvement of Krüppel‐like factor 4 (KLF4) in *miR*‐*145*‐dependent regulation of the phenotypic transformation of VSMCs in patients with AD under mechanical stretch conditions.

## 2 MATERIALS AND METHODS

### 2.1 Bioinformatics analysis of *miR*‐*145*

The GeneCards database was searched to analyze the position of *miR*‐*145* in the chromosome and the distributions of *miR*‐*145*, *miR*‐*221*, and *miR*‐*21* in different organs and tissues of the human body (https://www.genecards.org/). *miR*‐*145* regulates the proliferation and differentiation of smooth muscle cells by acting on target genes. We identified 9 target genes involved in these processes using PathCards (https://pathcards.genecards.org/). The String tool was used to draw a network diagram of the interaction between *miR*‐*145* and its target protein (https://string‐db.org/). The gene pathway related to the differentiation and proliferation of smooth muscle cells via *miR*‐*145* was found using NCBI (https://www.wikipathways.org/). Sites of interaction between *miR*‐*145* and KLF4 were analyzed using TargetScan (http://www.targetscan.org/).

### 2.2 Establishment of animal AD model

Thirty‐five healthy Sprague‐Dawley (SD) male juvenile rats (3 weeks old, weighing 65 ± 3 g each) were obtained from Shanghai Slack Laboratory Animal Co., Ltd. (permit no.: SCXK [Shanghai] 2017–0005). Animals were kept in a barrier environment at a temperature of 22–24°C with an alternating light/dark cycle (12 h/12 h) and adequate food and drinking water according to the experimental design. All test protocols complied with the guidelines for the use and protection of animals in laboratory (Institute of Laboratory Animal Resources, National Academy Press, Washington, DC, 1996), and the study was approved by the Experimental Animal Welfare Ethics Committee of Fujian Medical University.

Animals were randomly allocated to the experimental group (*n* = 25) or the control group (*n* = 10). β‐aminopropionitrile (BAPN) was dissolved in 2 ml drinking water for administration at 0.1 g/kg/day and administered by gavage once a day for 4 weeks. When rats were 7 weeks old, they were injected intraperitoneally with 1.44 mg/kg AngII dissolved in 0.5 ml, three times a day (at 8‐h intervals) for 1 week. The state of the rats was closely observed. If there was a sudden death during the test, the rat was immediately subjected to necropsy using a microscope (CX41; Olympus, Japan) to observe AD formation. If AD was found, the aortic vessels were removed, and the tissues were immediately frozen in liquid nitrogen and stored in a −80°C freezer or fixed in 10% formalin for paraffin embedding.

### 2.3 Hematoxylin‐eosin (HE) staining

Samples had been collected, dehydrated in ethanol solution, and transparentized in dimethylbenzene, which were cut into slices after paraffin embedding. The slices were baked, dewaxed, hydrated, and incubated in hematoxylin solution for 3 min, which were then differentiated in alcoholic hydrochloric acid for 15 s and blued in the Scott bluing to buffer for another 15 s. Next, the slices were washed and incubated in eosin solution for 3 min. Finally, they were dehydrated, transparentized, mounted, and observed under a microscope (CKX41, Olympus, Japan).

### 2.4 Victoria blue staining

Samples had been collected, dehydrated in ethanol solution, and transparentized in dimethylbenzene, which were cut into slices after paraffin embedding. The slices were incubated in Weigert solution for 10 min, differentiated in alcoholic hydrochloric acid for 10 s, and blued in Victoria blue elastic fiber dyeing buffer for 4 min. By finishing the previous procedures, the slices were washed and incubated in ponceau S‐fuchsin solution for 7 min. Subsequently, they were washed with weak acid solution and phosphomolybdic acid solution before staining in aniline blue solution for 1 min. Finally, they were dehydrated, transparentized, mounted, and observed under a microscope (CKX41, Olympus, Japan).

### 2.5 Clinical samples

Patients with acute AD and those who underwent heart transplantation at the Union Hospital of Fujian Medical University (Fuzhou, China) from December 2018 to December 2019 were enrolled in this study. Acute aortic wall samples (*n* = 15) and aortic wall samples from heart transplant donors (*n* = 6) were obtained, frozen immediately in liquid nitrogen, and stored in a −80°C freezer or fixed in 10% formalin for paraffin embedding. All samples were collected after obtaining informed consent from the patients, and the study was approved by the Institutional Review Committee and regulatory authorities of Fujian Medical University (2018KJT091).

### 2.6 Extraction, cultivation, and identification of VSMCs from patients with aortic dissection

In patients with acute AD, diseased aortic wall samples were collected, and the intima of the blood vessels was fully peeled off with a scalpel and trimmed into 2 × 2 mm tissue blocks. The tissue blocks were spaced 1 cm apart to cover the bottom of a 25T cell culture flask, and flasks were then placed in an incubator (5% CO_2_, 37°C) for 30 min. An appropriate amount of smooth muscle cell culture medium (SMCM) was added to the culture. The cells migrated out of the tissue block within 5–6 days, and cell growth and fusion reached 90% after 2 weeks. VSMCs were purified using the differential adhesion method. At passages 3–4, cells were showed to have a high purity by immunohistochemistry (α‐SMA).

### 2.7 Immunohistochemistry

4% paraformaldehyde treatment for 30 min, PBS rinse for 6 min; 0.5% Txiton X‐100 treatment for 20 min, PBS rinse for 6 min; 3% H_2_O_2_ treatment for 15 min, PBS rinse for 6 min; serum blocking, 37°C, 20 min; anti‐α‐SMA antibodies (Abcam, Cambridge, UK; cat. no. ab5694), 37°C, 2 h, PBS rinse for 9 min; goat anti‐rabbit antibody (Thermo Fisher, cat. no. A16096), 37°C, 30 min, PBS rinse for 9 min; SABC (Solarbio, cat. no. SA0022), 37°C, 20 min, PBS rinse for 9 min; DAB (Solarbio, cat. no. SW1020) color development, PBS rinse for 2 min; hematoxylin counterstain for 2 min, Rinse with PBS for 30 min, dehydration with 75%‐85%‐95%‐100% gradient alcohol for 5 min; xylene is transparent for 6 min, sealed with neutral gum, and observed under microscope.

### 2.8 Cell stretch application

AD‐VSMCs were inoculated on Flexcell 6‐well plates coated with collagen. When the AD‐VSMCs reached 80–90% fusion, serum‐free SMCM was added, and cells were cultured for an additional 24 h. The Flexcell 6‐well plate was installed on a negative pressure tension plate, the air compressor was applied, and different tensions (0%, 10%, 20%, and 30%) were applied. Briefly, the stretch system controls the negative pressure to make the silicone membrane at the bottom of the stretch plate deform, which generates the corresponding mechanical tension in cells attached to the silicone membrane. Next, AD‐VSMCs were collected, total protein was extracted.

### 2.9 Western blot analysis

RIPA lysis buffer (strong) RIPA (YEASEN, 20101ES60, USA) was used to extract proteins from mechanically stretched and unstretched AD‐VSMCs. The protein concentration was determined using the BCA method, and Western blotting was then used to detect protein levels. Briefly, protein extracts were separated by sodium dodecyl sulfate polyacrylamide gel electrophoresis and transferred to polyvinylidene difluoride membranes (Millipore, USA). The membranes were then blocked with 5% skim milk for 2 h and incubated with primary antibodies overnight at 4°C. After washing three times with TBST, the membranes were incubated with secondary antibodies for 1 h. Visualization was performed using an enhanced chemiluminescence detection system, and Photoshopcs3 software was used to analyze the relative band intensity. The primary antibodies used in this study were as follows: anti‐α‐SMA antibodies (Abcam, Cambridge, UK; cat. no. ab7817), anti‐SM22‐α antibodies (Abcam; cat. no. ab14106), anti‐OPN antibodies (Abcam; cat. no. ab8448), anti‐PCNA antibodies (Abcam; cat. no. ab29), anti‐KLF4 (Abcam; cat. no. 106629), anti‐MMP9 antibody (Abcam; cat. no. ab76003), anti‐GAPDH antibody (Abcam; cat. no. ab9485) and anti‐β‐actin antibody (Arigo; cat. no. arg62346).

### 2.10 Real‐time quantitative polymerase chain reaction (RT‐qPCR)

To detect the expression of miRNAs, including *miR*‐*145*, we performed RT‐qPCR using universal cDNA synthesis and SYBR Green Master Mix (Denmark). *U6* rRNA expression was used as an internal control. The specific RNA PCR primer set and U6 primers were obtained from Exiqon (Denmark).

To detect levels of *pri*‐*miR*‐*145*, total RNA was collected using a PrimeScript RT kit (Takara, Japan) and reverse transcribed into cDNA. Real‐time PCR was then performed using SYRB Premix Ex Taq kit (Japan). The PCR cycle conditions were as follows: 95°C for 3 min, 95°C for 40 s, 56°C for 10 s, 40 cycles, and 72°C for 10 s. The primer sequences used were as follows: *hsa*‐*miR*‐*145*‐*5p*‐F, CCGGTCCAGTTTTCCCAGGA; *hsa*‐*miR*‐*145*‐*5p*‐R, AGTGCAGGGTCCGAGGTATT;

*hsa*‐*miR*‐*145*‐*5p*‐RT, GTCGTATCCAGTGCAGGGTCCGAGGTATTCGCACTGGATACGACAGGGAT.

### 2.11 Transfection of AD‐VSMCs

Transfection of AD‐VSMCs was performed with *hsa*‐*miRNA*‐*145*‐*5p* mimic (Shanghai Genechem Co., Ltd.) to upregulate and downregulate *hsa*‐*miRNA*‐*145*‐*5p* in AD‐VSMCs. The sequences for *hsa*‐*miRNA*‐*145*‐*5p* mimic, and *hsa*‐*miRNA*‐*145*‐*5p* mimic control were as follows: *hsa*‐*miRNA*‐*145*‐*5p* mimic sense, GUCCAGUUUUCCCAGGAAUCCCU; *hsa*‐*miRNA*‐*145*‐*5p* mimic antisense, AGGGAUUCCUGGGAAAACUGGAC; *hsa*‐*miRNA*‐*145*‐*5p* mimic control sense, UUCUCCGAACGAGUCACGUUU; *hsa*‐*miRNA*‐*145*‐*5p* mimic control antisense, ACGUGACUCGUUCGGAGAAUU, *hsa‐miRNA‐145‐5p inhibitor*, AGGGAUUCCUGGGAAAACUGGAC; *hsa*‐*miRNA*‐*145*‐*5p inhibitor control*, ACGUGACUCGUUCGGAGAAUU.

Transfection was performed as follows. Briefly, AD‐VSMCs were inoculated into 6‐well plates and cultured until the cell density reached 80–90%. The medium was changed to serum‐free medium and cultured for an additional 2 days to maintain AD‐VSMCs in the logarithmic growth phase. For transfection, the samples were prepared as follows. First, 125 µl serum‐free SMCM, 100 pmol siRNA (a 5‐Fam fluorescent transfection indicator was added to the indicator group), and 4 µl LipoRNA I transfection reagent were mixed gently and then incubated at room temperature for 20 min. The transfection mixture was added to each well of a 6‐well plate, and plates were incubated for 6 h at 37°C in an atmosphere containing 5% CO_2_. The medium was then incubated with serum‐free SMCM for 48 h.

### 2.12 Statistical analysis

Continuous variables were presented as means with SD or medians with IQR, and analyzed by analysis of variance (ANOVA) models or student's *t* test (two‐tailed) or rank‐sum test. Statistical analyses were performed using SPSS version 24.0 and a *p*‐value <0.05 was considered statistically significant.

## 3 RESULTS

### 3.1 Identification of the signaling pathway and PPI network through which *miR*‐*145* regulated the differentiation of VSMCs

**FIGURE 1:**
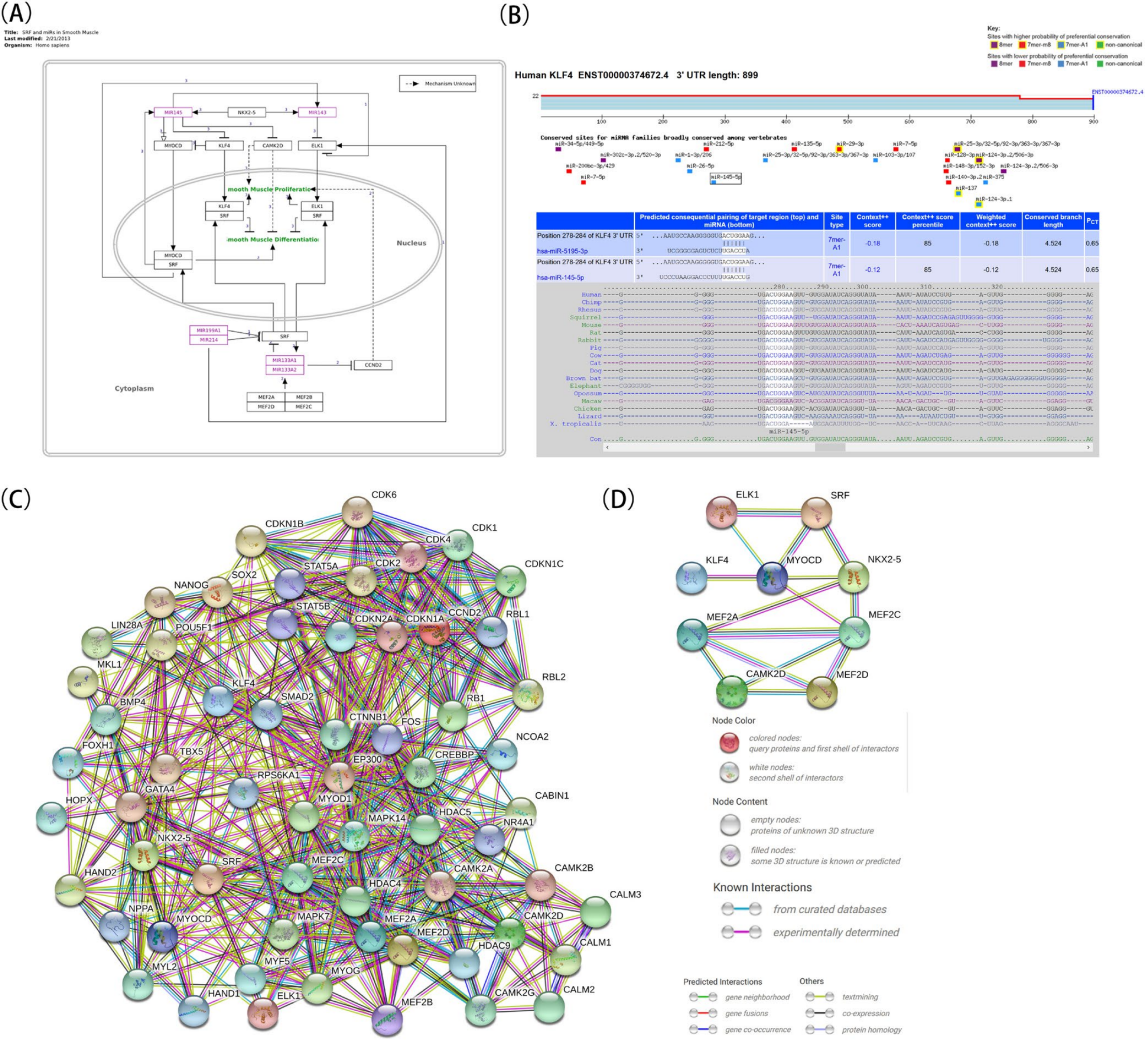
Bioinformatics analysis of regulators involved in phenotypic transformation of vascular smooth muscle cells. (A) The signaling pathway through which *miR*‐*145* regulated the proliferation and differentiation of VSMCs. (B) The binding site between *hsa*‐*miR*‐*145*‐*5p* and the 3′ untranslated region (UTR) of KLF4. (C) Protein‐protein interaction (PPI) network through which miR‐145 regulated VSMC differentiation. The PPI network was drawn using STRING online. Shows 61 protein nodes and 552 interaction relationships, with a minimum confidence level of 0.4 (moderate confidence). Disconnected nodes are hidden in the network. (D) Shows the first nine proteins involved in the regulation of VSMC differentiation with a minimum requirement of 0.7 (highly credible)

WikiPathways database showing that *miR*‐*145*/‐*143* mediated VSMC proliferation and differentiation by directly or indirectly acting on KLF4, ETS like‐1 protein (ELK1), myocardin (MYOCD), and serum response factor (SRF) (Figure 1A). We used the TargetScan tool to predict the binding site between *hsa*‐*miR*‐*145*‐*5p* and the 3′ untranslated region (UTR) of *KLF4* (Context++ score = −0.12, Context++ score percentile = 85, PCT = 0.65; Figure 1B). TargetScan did not predict any binding sites for *miR*‐*145* in *ELK1*, *MYOCD*, and *SRF*.

PPI networks are composed of nodes that represent proteins and edges that describe related interactions. A PPI network through which *miR*‐*145* regulated VSMC differentiation was established. This network contained 61 protein nodes and 552 interaction relationships, with a minimum confidence level of 0.4 (moderate confidence; Figure 1C). We screened out nine proteins involved in the regulation of VSMC differentiation (SRF, KLF4, MYOCD, ELK1, calcium/calmodulin‐dependent protein kinase IIδ [CAMK2D], NK2 homeobox 5, myocyte‐specific enhancer factor [MEF] 2A, MEF2C, MEF2D), with a minimum requirement of 0.7 (highly credible), and established the interaction diagram (Figure 1D).

### 3.2 Successful establishment of an AD model

**FIGURE 2:**
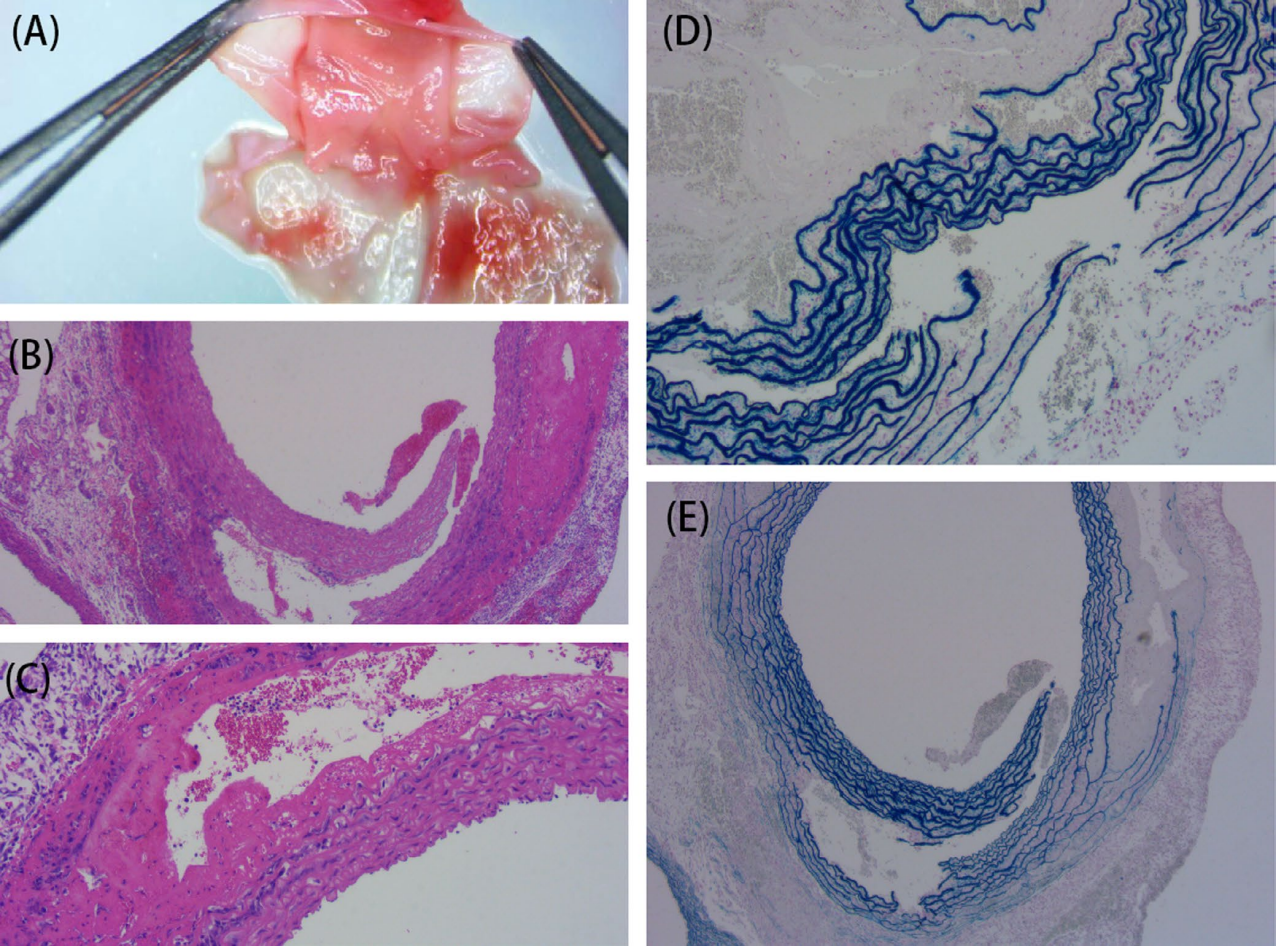
Pathological examination of aortae. (A) The anatomy of aorta in SD rats with AD (×10). (B, C) HE (×100, ×400) and (D, E) Victoria blue staining (×100, ×400) of aortae in the model rats. Victoria blue staining shows the broken elastic fibers in the aortic media

Among the 25 experimental rats, 17 died suddenly after the drug intervention. Necropsies showed that all causes of death were AD, and none of the surviving rats showed AD. The incidence of AD in our animal model was 68%. The blood vessels in SD rats with AD were dissected (Figure 2A) and stained with hematoxylin and eosin (Figure 2B and C) and Masson staining (Figure 2D and E). The structure of the aortic wall in the control group was complete and continuous. In AD rats, the aortic wall contained broken elastic fibers, and blood cells enter the wall, leading to tearing, peeling, and the formation of a false lumen. These results supported that the AD rat model was successfully established.

### 3.3 Differential expression of matrix metalloproteinase (MMP) 9 in rats

**FIGURE 3:**
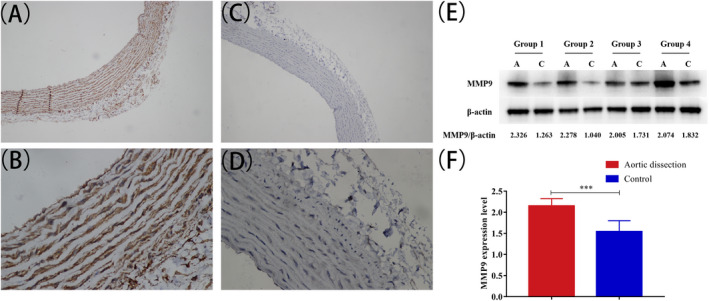
Expression levels of MMP9 in rat aortic vessel walls tissues. Immunohistochemical staining showing the expression of MMP9 in aortic vessel walls from rats with AD (A, B) (×100,×400) and NC (×100,×400) (C, D). (E, F) WB showing expression levels of MMP9 in aortic vessel walls from AD rats. Data are shown mean ± SEM. *p < 0.05, **p < 0.01, ***p < 0.001 vs control

Figure 3A‐D shows detection of MMP9 by immunohistochemical staining of aortic vessel walls from rats with AD and control group. We further analyzed the protein expression levels of MMP9 by Western blotting (Figure 3E and F). Compared with the normal group, the expression of MMP9 protein in aortic vessels of model rats was significantly increased. These results suggested that MMP9 secretion was increased in vascular wall cells under mechanical stretch caused by hypertension.

### 3.4 *miR*‐*145* expression was downregulated in model rats and patients with AD

**FIGURE 4:**
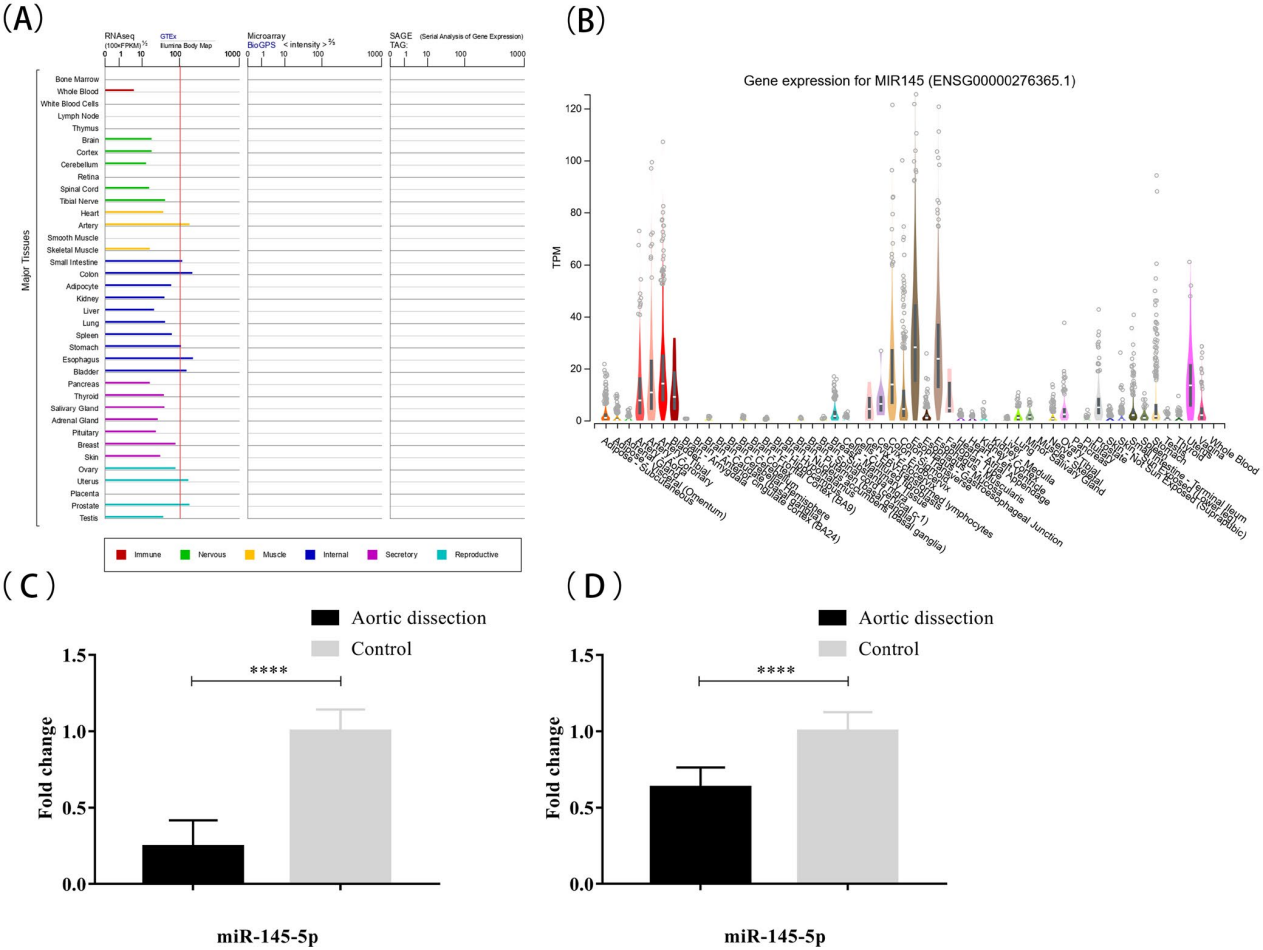
Expression of miR‐145 in vascular tissues. (A) The differences in the expression of miR‐145 in various tissues in the normal human body in GTEx database. Fragments per kilobase of transcript per million mapped reads were used as the standardized value, with 100 M reads used for segmentation. For tissues with more than 100 M reads, miR‐145 was considered highly expressed. The results showed that miR‐145 was abundantly expressed in the arteries, small intestine, colon, stomach, esophagus, bladder, uterus, and prostate. (B) The genotype‐tissue expression (GTEx) project was supported by the Common Fund of the Office of the Director of the National Institutes of Health, and by NCI, NHGRI, NHLBI, NIDA, NIMH, and NINDS. Data Source: GTEx Analysis Release V8 (dbGaP Accession phs000424.v8.p2). Real‐time qPCR showing miR‐145 levels in AD rats (C) and patients (D). Data are shown mean ± SEM of 4 experiments. *p < 0.05, **p < 0.01, ***p < 0.001 vs control

The GTEx and BioGPS databases from GeneCards showing the miR‐145 expression in normal human tissues. For tissues with more than 100 M reads, *miR*‐*145* was considered highly expressed (Figure 4A). The results showed that *miR*‐*145* was abundantly expressed in the arteries, small intestine, colon, stomach, esophagus, bladder, uterus, and prostate (Figure 4B).

RT‐qPCR showed that *miR*‐*145* was significantly downregulated in aorta samples from the model rats compared with that in the control group (Figure 4C). In order to further verify the effects of mechanical stretch caused by hypertension on *miR*‐*145* expression in the human aorta, we obtained aortic wall specimens from 10 patients with AD (experimental group) and 6 heart transplant donors (control group) and assessed *miR*‐*145* expression by RT‐qPCR. Compared with the control group, *miR*‐*145* expression was downregulated in the experimental group (Figure 4D). These findings suggested that mechanical stretch downregulated *miR*‐*145* in the aortic vessels of rats and humans with AD.

### 3.5 Mechanical stretch‐induced phenotypic transformation of VSMCs in model rats and patients with AD

**FIGURE 5:**
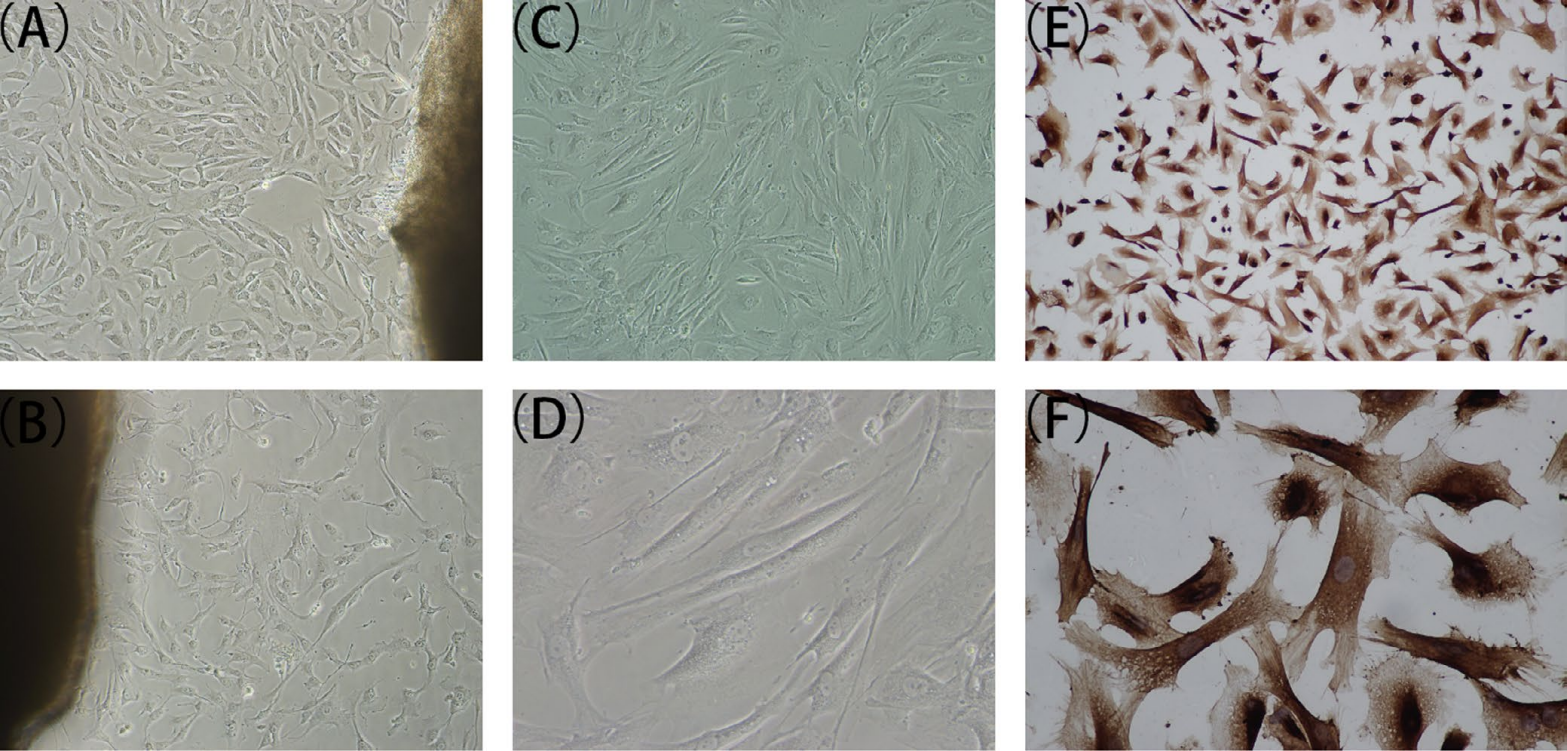
Cultivation and identification of AD‐VSMCs. (A, B) Primary AD‐VSMCs under a light microscope (×100). (C, D) AD‐VSMCs passaged 4–5 under a light microscope (×100, ×200). (E, F) Identification of purified smooth muscle cells by immunohistochemistry (α‐SMA).

From the diseased vascular tissues of patients with AD, we successfully separated tissues and cultured primary VSMCs (Figure 5A and B). Cells were purified and cultures (Figure 5C and D). The purified smooth muscle cells were identified by immunohistochemistry (Figure 5E and F) and showed a high purity.

**FIGURE 6:**
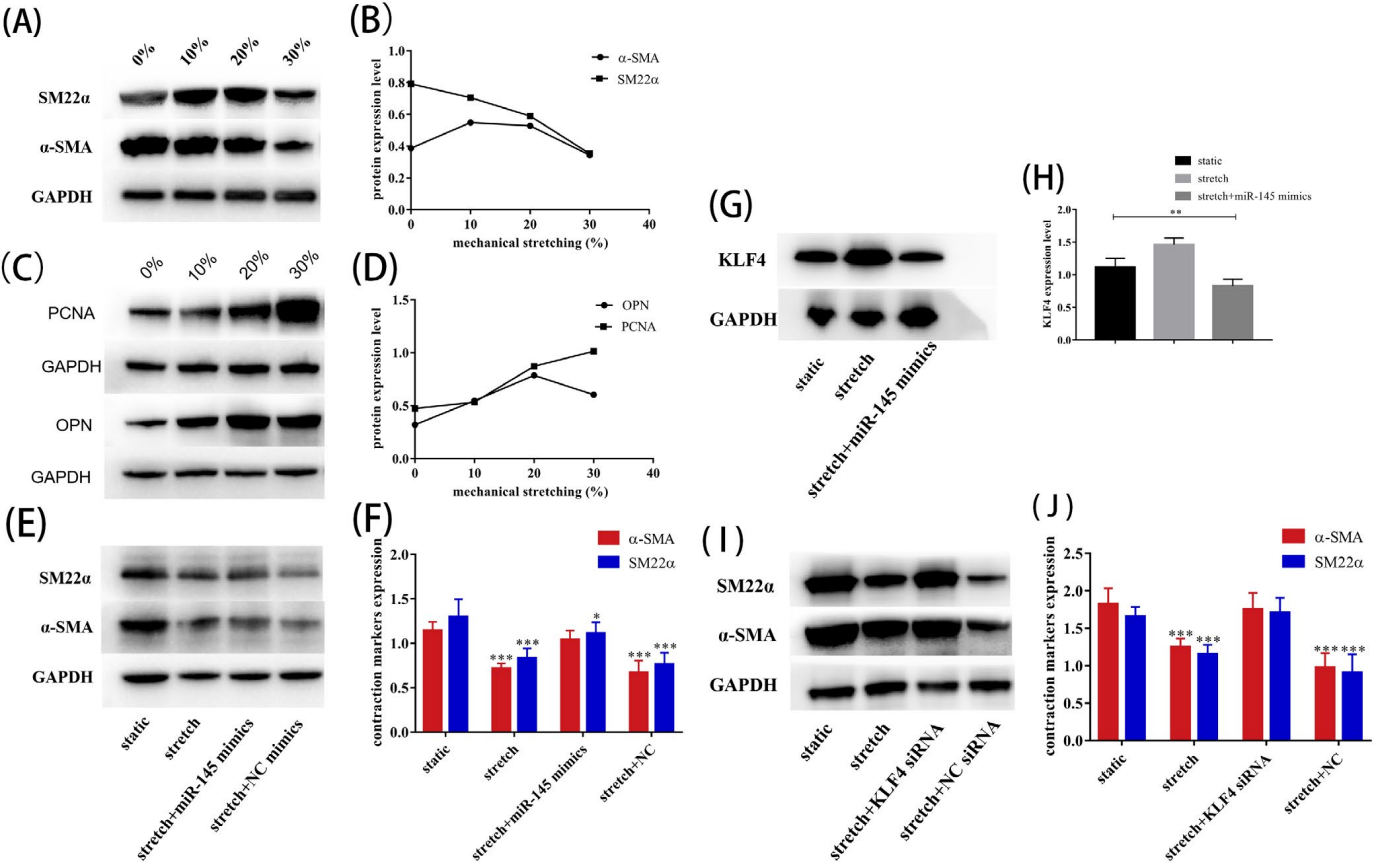
miR‐145 targeted KLF4 to participate in mechanical stretch‐induced phenotypic transformation of VSMCs. (A‐D) The VSMC contraction and secretion markers expression of AD‐VSMCs under different mechanical tension. The results showed that the expressions of VSMCs phenotype proteins α‐SMA, SM22α, OPN, and PCNA were statistically significant under different tensions. The results are as follows, α‐SMA *χ^2^* = 8.333 *p* = 0.040; SM22α *χ^2^* = 8.231 *p* = 0.041; OPN *χ^2^* = 8.897 *p* = 0.031; PCNA *χ^2^* = 8.744 *p* = 0.033. (E, F) Modulation of miR‐145 levels by use of miRNA mimics in AD‐VSMCs exposed to 20% stretch for 24 h. (G, H) Western blot analysis and quantification of protein levels of KLF4 protein level treated with miR‐145 mimics in stretched AD‐VSMCs and (I, J) Western blot analysis of protein level of VSMC contractile markers with KLF4 siRNA knockdown and negative control (NC) siRNA knockdown under stretch treatment Data are mean ± SEM of 4 experiments. *p < 0.05, **p < 0.01, ***p < 0.001 vs static

Purified AD‐VSMCs (passage 4) were placed in serum‐free SMCM for 24 h, and smooth muscle cells were stretched for 24 h by applying different traction tensions in smooth muscle cells. Compared with control cells, AD‐VSMCs subjected to 30% mechanical tension showed decreased expression of VSMC contraction markers, including α‐SMA, SM22, and the expression levels of markers of contraction were also low (Figure 6A and B). In contrast, the expression levels of the VSMC secretion markers PCNA and OPN were higher under mechanical stretching than under static culture. The expression of secretion marker proteins increased after application of mechanical stretching, and expression levels increased as mechanical tension increased (Figure 6C and D).

### 3.6 *miR*‐*145* participated in the phenotypic transformation of VSMCs in patients with AD following mechanical stretching

Compared with static cultured cells, the expression levels of the contraction marker proteins α‐SMA and SM22α were reduced in AD‐VSMCs under mechanical stretching, whereas the expression levels of the marker proteins PCNA and OPN secreted by VSMCs were increased. To further verify whether *miR*‐*145* was involved in this process, we used gain‐of‐function methods to evaluate the effects of altered *miR*‐*145* expression on the phenotype of AD‐VSMCs. Our results showed that overexpression of *miR*‐*145* significantly reduced the expression of stretch‐inhibiting contraction markers compared with that in cells subjected to mechanical stretch (Figure 6E and F).

### 3.7 *miR*‐*145* targeted KLF4 to participate in mechanical stretch‐induced phenotypic transformation of VSMCs

Compared with statically cultured AD‐VSMCs, after applying mechanical stretch, the expression of KLF4 protein was significantly increased in AD‐VSMCs. However, upregulation of KLF4 under mechanical stretching was inhibited by the *miR*‐*145* mimic (Figure 6G and H). Notably, after knockdown of KLF4 by siRNA in AD‐VSMCs, KLF4 expression was inhibited. Under mechanical stretching, the expression of the contraction markers of AD‐VSMCs was increased compared with that in the control group (Figure 6I and J).

## 4 DISCUSSION

Surgical operation is the only approach for curing AD; however, the surgery is invasive and costly, and mortality rates are still high, even after treatment. Therefore, it is necessary to identify biological markers and therapeutic targets in order to improve outcomes in patients with AD.

Hypertension is closely related to the occurrence of AD. Because of the features of blood flow and blood pressure,^12^, ^13^ blood vessels are constantly exposed to mechanical forces in the form of shear stress and stretching, and VSMCs begin to differentiate from the contraction type to the secretion type.^14^, ^15^ Under normal physiological conditions, VSMCs in the middle layer of the aorta are mainly contractile; then, under induction of mechanical tension caused by hypertension, VSMCs begin to transform into the secretory type.^16^, ^17^ AD‐VSMCs also show similar results under stretching. Indeed, in this study, the phenotype of AD‐VSMCs changed before and after stretching. The expression levels of the contractile markers α‐SMA and SM22α decreased after mechanical stretching, whereas those of the secretion markers PCNA and OPN increased. Secreted VSMCs secrete more MMPs, destroying the original physiological structure of the aortic wall, degrading the elastic fibers in the middle of the aorta, and increasing the risk of AD.^18^, ^19^ Importantly, VMSCs are known to secrete increased levels of MMP9, which in turn degrades the extracellular matrix, remodels the vascular wall, and induces the formation of AD.^20^, ^21^, ^22^ We found that MMP9 expression was included in model rats. Thus, our findings showed that mechanical stretch induced the differentiation of VSMCs into the secretory type.

Mechanical stretch simulates the effects of hypertension on the blood vessel wall. The blood pressure in the human body fluctuates dynamically. However, transformation of AD‐VSMCs under different mechanical tensions has not yet been reported. In this study, we assessed the effects of different tensions and showed that the VSMC contraction marker proteins α‐SMA and SM22α were negatively correlated with the magnitude of mechanical tension. Indeed, after static culture of AD‐VSMCs subjected to mechanical tension, the expression levels of contractile markers were reduced. The decreasing trend became more obvious as the tension increased. Additionally, PCNA and OPN expression levels depended on force and increased as the mechanical tension increased, showing a positive correlation. These findings supported that mechanical stretch‐induced phenotype changes in AD‐VSMCs and that the degree of cell differentiation was positively correlated with the magnitude of mechanical stretch.

The miRNA expression profiles of *miR*‐*221*, *miR*‐*21*, and *miR*‐*145* are significantly altered in human aortic smooth muscle cells after exposure to mechanical tension. Indeed, in this study, we identified *miR*‐*221* and *miR*‐*145* in the GTEx database and *miR*‐*21* in normal human tissues and organs in the BioGPS database. *miR*‐*221* expression in arterial vascular tissues was not significantly different from that in other tissues, and *miR*‐*21* showed weak expression in arterial vascular tissues. In contrast, *miR*‐*145* expression was abundant in the aorta, arteries, small intestine, colon, stomach, esophagus, bladder, uterus, and prostate. In previous studies, *miR*‐*145* has been shown to be the most abundant miRNA in the arteries and to be significantly downregulated in blood vessel walls after injury to the vascular intima.^23^ These miRNAs are mainly expressed in blood vessels and other tissues and organs that are rich in smooth muscle cells, and their absence leads to incomplete differentiation of VSMCs.^24^ Smooth muscle cells exhibit unique plasticity because they can switch between a proliferative state (secreted type) and a more stable, highly differentiated state (contracted type). These two states are partly determined by a network of transcription factors, including KLF4, ELK1, and SRF. The expression levels of these genes affect the state of smooth muscle cells. The positive feedback of *miR*‐*145* enhances the expression of the smooth muscle regulatory factor MYOCD, which cooperates with SRF to activate *miR*‐*145* transcription. Additionally, *miR*‐*145* then coordinately targets this transcription factor network and regulates the differentiation of VSMCs.

Changes in *miR*‐*145* expression also affected the differentiation of VSMCs, and this miRNA was significantly downregulated in the aortic vascular wall tissues of model rats, patients with AD, and AD‐VSMCs after application of mechanical tension. Moreover, mechanical stretching induced the differentiation of AD‐VSMCs. Our analysis demonstrated that *miR*‐*145* mediated the differentiation of VSMCs and may be involved in the mechanical stretch‐induced phenotypic transformation of AD‐VSMCs. Indeed, overexpression of *miR*‐*145* decreased the expression of secretory phenotype markers and increased the expression of contraction markers, suggesting that *miR*‐*145* blocked the transformation of AD‐VSMCs from the contractile to the secretory type. Overall, these findings suggested that *miR*‐*145* could effectively inhibit the differentiation of AD‐VSMCs under mechanical stretching.

Finally, we showed that *miR*‐*145* participated in the regulation of VSMC differentiation via modulation of KLF4 expression to participate in the regulation of VSMC differentiation. Importantly, KLF4 inhibits the expression of contractile proteins in VSMCs by interacting with SRF.^25^ However, the induction of KLF4 expression by mechanical stretch has not been clarified. Our current results suggested that mechanical stretch increased KLF4 protein expression, accompanied by downregulation of *miR*‐*145*. Overexpression of *miR*‐*145* inhibited KLF4 protein expression, suggesting that KLF4 was negatively correlated by *miR*‐*145*. Additionally, mechanical stretch downregulated *miR*‐*145* expression by activating the stretch pathway, and downregulation of *miR*‐*145* further stimulated KLF4 expression, which induced AD‐VSMC differentiation.

Despite the strength of our findings, there were some limitations of this study. For example, we only analyzed the relationships of mechanical stretch with *miR*‐*145* and KLF4 at the cell level in vitro in order to avoid the interference of other factors, such as inflammatory factors, in patients with AD. We analyzed phenotype changes in AD‐VSMCs after exposure to different stretch intensities and assessed the expression patterns of *miR*‐*145* under different stretch conditions to determine the relationships between *miR*‐*145* and mechanical tension. However, additional studies are required to confirm and further expand upon our findings.

In summary, our findings showed that KLF4 may be a potential therapeutic target in patients with AD. We also showed that *miR*‐*145* inhibited the differentiation of AD‐VSMCs by increasing KLF4 degradation or reducing KLF4 expression; these changes delayed the progression of hypertension‐induced aortic wall remodeling and further suppressed the occurrence of AD.

## CONFLICT OF INTEREST

The authors have no conflicts of interest to disclose.

## Data Availability

The data that support the findings of this study are available from the corresponding author upon reasonable request.
